# Pain and rehabilitation after total hip arthroplasty are approach dependent: a multisurgeon, single-center, prospective cohort study

**DOI:** 10.1007/s00402-021-03921-0

**Published:** 2021-05-08

**Authors:** Alexander Zimmerer, Mona Steinhaus, Erdmann Sickmüller, Benjamin Ulmar, Matthias Hauschild, Wolfgang Miehlke, Stefan Kinkel

**Affiliations:** 1 ARCUS Sportklinik Pforzheim, Rastatterstr. 17-19, 75179 Pforzheim, Germany; 2 Department of Orthopedics and Orthopedic Surgery, University Medicine Greifswald, Ferdinand-Sauerbruch-Straße, 17475 Greifswald, Germany

**Keywords:** Total hip replacement, Rehabilitation, PROM, Anterior approach, Lateral approach, Posterior approach

## Abstract

**Purpose:**

The aim of this study was to assess perioperative pain and mobilization after total hip arthroplasty (THA) using three different surgical approaches.

**Methods:**

This was a multisurgeon, prospective, single-center cohort study. A total of 188 patients who underwent hip arthroplasty (THA) between February 2019 and April 2019 were analyzed according to the surgical approach used (direct anterior, lateral, and posterior approach). Outcome parameters were the daily walking distance during the inpatient stay, the pain level according to the visual analog scale (VAS) at rest and motion during the inpatient stay and at 6-week follow-up and the modified Harris Hips Score (mHHS) preoperatively and at 6 weeks.

**Results:**

The walking distance within the groups increased significantly during the inpatient stay (*p* < 0.001). The DAA and posterior approach patients had a significantly longer walking distance than the lateral approach patients on the third postoperative day (DAA vs. lateral, *p* = 0.02; posterior vs. lateral 3, *p* = 0.03). DAA and posterior approach patients reported significantly less pain during motion on the third postoperative day and at 6-week follow-up than the lateral approach patients (3 postoperative day: DAA vs. lateral, *p* = 0.011; posterior vs. lateral, *p* = 0.04; 6 weeks control: DAA vs. lateral, *p* = 0.001; Posterior vs. lateral 3, *p* = 0.005). The mHHS demonstrated significant improvement within each group. However, lateral approach patients reported significantly less improvement than the DAA and posterior approach patients (DAA vs. lateral, *p* = 0.007; posterior vs. lateral, *p* = 0.021).

**Conclusion:**

This study analyzed perioperative pain progression and short-term rehabilitation after THA according to the different surgical approaches. Direct anterior and posterior approaches have shown comparable improvements in pain, walking distance, and mHHS. Whether this effect persists over a longer period of time must be clarified in future studies.

**Study design:**

Prospective cohort study, level of evidence, 2.

## Background

Coxarthrosis is a common disease of the hip joint and it is typically treated by total hip arthroplasty (THA) if conservative therapy is not successful. The majority of patients undergoing THA experience pain relief, improved mobility, enhanced functionality and the restoration of the quality of life [1, 2]. Moreover, several studies have shown improved patient-reported outcome measures (PROMs) following THA [3, 4]. Nevertheless, a small minority of THA patients continue to suffer from symptoms, mostly pain, that prevent their return to full function and activity. Possible underlying causes include fixation failure, instability, and soft tissue damage, related to the trauma of the surgical procedure. The latter seems to have a major influence on the short-term results due to soft tissue damage [5–7].

The three most common methods used for THA are the anterior, lateral, and posterior approaches [8]. However, there is still no consensus on the effect of the surgical approach on postoperative pain. In recent years, many studies have compared two of these approaches (anterior vs. posterior, anterior vs. lateral, posterior vs. lateral) [5, 9–15]. In these studies, the anterior approach appears to be superior to the other approaches in terms of postoperative pain and short-term functional outcome. To the best of our knowledge, only a few studies have investigated and compared all three approaches against each other according to their effect on postoperative pain after THA [8, 16, 17]. Therefore, the aim of this study was to assess perioperative pain and mobilization after THA in relation to the three different surgical approaches. We hypothesized that DAA patients will report less pain and mobilize more quickly than patients who receive the lateral or posterior approach.

## Methods

The present study comprises a consecutive series of 188 patients following cementless total hip arthroplasty performed in a single-center, multisurgeon series (7 surgeons) between February 2019 and April 2019. Patients were evaluated prospectively over the study period. The Corail Pinnacle Hip System (DePuy Synthes, Warsaw, IN) was used in all patients. Three groups were formed according to the approach used: Group 1, minimally invasive direct anterior approach (DAA); Group 2, transgluteal lateral approach; Group 3, piriformis muscle sparing posterior approach. Each surgeon performs THA with only one approach at high volumes (> 250/year). Thus, every patient of the respective surgeon was operated on using the same approach. The inclusion criteria consisted of primary cementless total hip arthroplasty and consent for general anesthesia. Exclusion criteria included revision surgery, the existence of chronic pain syndrome preoperatively or for the use of epidural anesthesia (spinal block) to eliminate the bias of prolonged analgesia due to epidural anesthesia.

A standardized pain management concept was applied for all patients: Preoperatively the patients received 90-mg etoricoxib, 50-mg pregabalin and 20-mg pantoprazole. During surgery, dexamethasone at a dosage of 0.1–0.2 mg/kg, ondansetron 4 mg and tranexamic acid 500 mg were administered intravenously. A propofol bolus of 1.5–2.5 mg/kg was used intravenously to induce anesthesia. In addition, fentanyl 0.2 mg and the muscle relaxant rocuronium 0.5–0.6 mg/kg, which is monitored and controlled by relaxometry during surgery, were injected. Anesthesia was maintained by a continuous propofol infusion device. Postoperative oral-controlled analgesia was applied at the ward. The patients received 500 mg of metamizole four times daily and 90 mg of etoricoxib once daily as the standard. During the first 2 days, the patients received an additional 10-mg Targin in the morning and evening. During pain peaks, 10-mg Sevredol was administered every 4 h as needed.

Full weight-bearing was allowed in the immediate postoperative course. The initial mobilization of the patients was performed on the day of the operation as a standard, and patients also received physiotherapeutic treatment once a day from the day of the operation onwards. After discharge, pain-adapted full weight-bearing was further permitted. During the first 4 weeks, flexion over 90° and terminal rotational and adduction movements were prohibited. Ossification prophylaxis was given during the first 2 weeks, and thrombosis prophylaxis was given during the first 4 weeks postoperatively. The postoperative rehabilitation protocol was the same for all surgeons.

As a matter of routine, patients were prospectively assessed preoperatively and at 6 weeks postoperatively using the modified Harris Hip Score (mHHS) [18, 19]. The pain level was evaluated using a visual analog scale (VAS) for pain (VAS 0 = no pain; VAS 10 = worst pain imaginable) and was recorded preoperatively and on each day of hospitalization for rest and motion. It was also recorded 6 weeks postoperatively. The time of the initial mobilization as well as the daily walking distance were documented during the inpatient stay. In addition, factors such as depression, chronic pain syndrome, and diabetes that have been identified in the past as negative prognostic factors [20–23] for postoperative outcomes were documented. All data were collected daily by one investigator (M.S).

The ethics commission of the Landesaerztekammer Baden-Wuerttemberg, Germany approved all procedures (F-2019-006), and the study was conducted in accordance with the Helsinki Declaration of 1975, as revised in 2008. The study was registered in the German Registry of Clinical Studies (DRKS) with the approval number DRKS00016519 (WHO Register). All participants provided written informed consent.

### Statistics

G*Power [24] (version 3.1.9.2, 2014) was used for the sample size calculation. Assuming 5% as the acceptable margin of error, it was estimated that a total sample size of 180 patients would have 80% power in the study. Descriptive statistics for all continuous variables are reported as the means ± standard deviations. Differences between preoperative and postoperative data were examined with a *t* test and a Wilcoxon signed-rank test. Differences between the groups were tested using ANOVA test with a Bonferroni correction for multiple repeated comparisons. Categorical variables were reported using the count and percentage. To compare percentages between groups, Fisher’s exact test was performed. Statistical analyses were conducted using SPSS statistical software (IBM SPSS Statistics for Windows, version 26.0.0; IBM Corp).

## Results

### Demographics

**Fig. 1:**
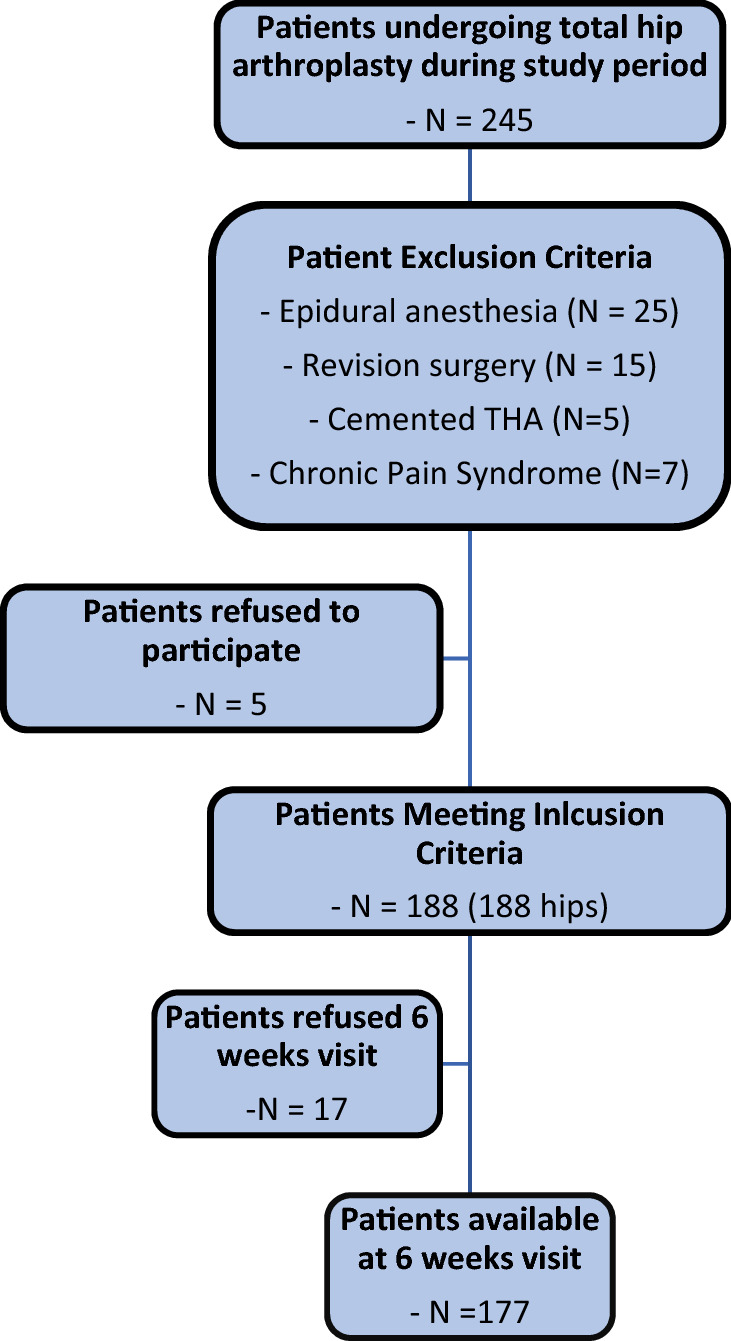
Flowchart illustrating the number of patients excluded from the study, lost to follow-up, and those who met the inclusion criteria

A total of 188 patients met the inclusion criteria and were included in the analysis (Fig. 1). There were 53% male hips (100/188) included. The mean patient age was 61.2 ± 10.2 (30.0–83.0) years, the mean body mass index (BMI) was 27.1 ± 4.6 (17.1–38.6) kg/m^2^, and the mean ASA score was 2.0 ± 0.6 (1.0–3.0) points. The percentages of negative prognostic factors (diabetes, depression, chronic pain syndrome) did not differ significantly among the groups. The proportions of patients who underwent arthroplasty of the contralateral hip or knee joint did not differ significantly. Ninety-eight THAs were performed on the right side, and 90 were performed on the left side. A total of 132 patients were discharged on the fourth postoperative day, and 56 were discharged on the fifth postoperative day. A significant difference was found among the three groups regarding the respective operation time (*p* < 0.006, ANOVA). In the post hoc pairwise comparisons (Bonferroni correction), we found statistically significant differences between groups 1 and 2 (*p* = 0.003) and groups 2 and 3 (*p* = 0.0019). No significant differences were found when comparing groups 1 and 3 (*p* = 0.356). Table 1 shows the data for each approach group.

**Table 1:** Patient demographic data according to the different approaches

Approach	DAA	Lateral	Posterior	*p* value
Total no. of patients	88	26	74	
Laterality, *n* (%)				
Right	48 (55)	14 (54)	36 (49)	0.832
Left	40 (45)	12 (46)	38 (51)	0.793
Sex, *n* (%)				
Male	42 (48)	14 (54)	44 (59)	0.421
Female	46 (52)	12 (46)	30 (41)	0.391
Age, y	61.9 ± 9.8 (30.0–81.0)	60.3 ± 12.3 (32.0–83.0)	60.7 ± 9.7 (31.0–81.0)	0.798
Body mass index, kg/m^2^	26.9 ± 4.1 (19.2–38.0)	27.0 ± 4.8 (17.1–36.3)	27.2 ± 4.9 (20.8–38.6)	0.832
ASA Score	2.0 ± 0.5 (1.0–3.0)	1.9 ± 0.6 (1.0–3.0)	1.9 ± 0.5 (1.0–3.0)	0.865
Negative prognostic comorbidities *n* (%)				
Diabetes	6 (7)	2 (8)	5 (7)	0.986
Depression	4 (5)	1 (4)	4 (5)	0.940
Chronic Pain Syndrome	2 (2)	0 (0)	2 (3)	0.708
Previous arthroplasties *n* (%)				
THA of the contralateral side	12 (14)	4 (15)	12 (6)	0.897
TKA	7 (8)	2 (8)	5 (7)	0.958
Preoperative pain medication *n* (%)				
NSAID	66 (75)	19 (73)	53 (72)	0.134
Opioids	8 (9)	2 (8)	5 (7)	0.860
Use of walking aids *n* (%)				
One walking cane	1 (1)	0 (0)	1 (1)	0.843
Two crutches	0 (0)	0 (0)	1 (1)	0.461

Values are shown as *n* (%), respectively as the mean ± SD (range)

*ASA* American Society of Anesthesiologists; *DAA* direct anterior approach; *NSAID* nonsteroidal anti-inflammatory drugs

### Postoperative mobilization

**Fig. 2:**
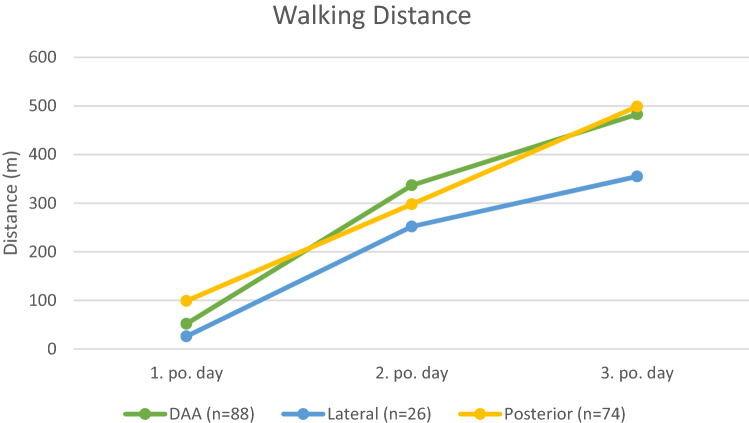
Presentation of the walking distances for the individual groups; DAA, direct anterior approach

A total of 185 (98%) patients could be mobilized on the day of surgery. In 3 (2%) patients, the first mobilization was only on the first postoperative day. The reason for the delay in these patients was increased nausea and circulatory weakness. The walking distance within the individual groups increased significantly from day to day (*p* < 0.001) (Fig. 2). Comparing the walking distance between the different groups, no significant differences were found for the first 2 postoperative days, with patients operated on using the lateral approach tending to walk a shorter distance. There was a significant difference on the third postoperative day. Patients in groups 1 and 3 had a significantly longer walking distance than those in group 2 (group1 vs. group 2, *p* = 0.02; group 2 vs. group 3, *p* = 0.03). There was no difference between groups 1 and 3 (*p* = 0.832).

### Pain Scores

Pain was assessed preoperatively, as well as daily during the hospital stay and for 6 weeks of follow-up at rest and during motion. The pain level decreased significantly over time in each group (*p* < 0.001). However, there were differences regarding the different points of time. DAA patients reported significantly less pain at rest at 6 weeks than in the lateral and posterior approach groups (group 1 vs. group 2, *p* = 0.004; group 1 vs. group 3, *p* = 0.007, group 2 vs. group 3, *p* = 0.371). There were no differences among the groups during the inpatient stay for the pain level at rest.

DAA and posterior approach patients reported significantly less pain during motion on the third postoperative day and at 6 weeks than the lateral approach group (third postoperative day: group 1 vs. group 2, *p* = 0.011; group 1 vs. group 3, *p* = 0.563, group 2 vs. group 3, *p* = 0.04; 6 weeks: group 1 vs. group 2, *p* = 0.001; group 1 vs. group 3, *p* = 0.300, group 2 vs. group 3, *p* = 0.005) (Table 2).

**Table 2:** Pain levels within the approach groups at the different time points

Approach	Preoperative	First postoperative day	Second postoperative day	Third postoperative day	6 weeks postoperative
	VAS pain for rest				
DAA	4.7 ± 2.8 (0.0–10.0)	1.3 ± 1.5 (0.0–8.0)	1.1 ± 1.3 (0.0–6.0)	0.8 ± 1.1 (0.0–6.0)	0.2 ± 0.5 (0.0–2.0)^a^
Lateral	3.5 ± 3.1 (0.0–10.0)	1.8 ± 1.6 (0.0–6.0)	1.3 ± 1.5 (0.0–6.0)	1.1 ± 1.5 (0.0–6.0)	0.6 ± 0.8 (0.0–3.0)^a^
Posterior	4.6 ± 3.0 (0.0–10.0)	1.7 ± 1.5 (0.0–6.0)	1.2 ± 1.1 (0.0–4.0)	1.0 ± 1.1 (0.0–4.0)	0.6 ± 1.1 (0.0–4.0)^a^
	VAS pain for motion				
DAA	7.3 ± 2.0 (2.0–10.0)	4.8 ± 2.2 (0.0–10.0)^b^	3.8 ± 2.1 (0.0–9.0)	3.0 ± 2.0 (0.0–8.0)^c^	1.1 ± 1.1 (0.0–4.0)^c^
Lateral	7.4 ± 2.0 (3.0–10.0)	5.9 ± 2.4 (1.0–10.0)^b^	4.6 ± 2.9 (0.0–10.0)	4.4 ± 2.7 (0.0–10.0)^c^	2.1 ± 1.4 (0.0–6.0)^c^
Posterior	6.5 ± 2.2 (0.0–10.0)	5.2 ± 2.2 (1.0–10.0)	4.0 ± 1.9 (1.0–8.0)	3.1 ± 1.8 (0.0–8.0)^c^	1.3 ± 1.3 (0.0–6.0)^c^

Presentation of the pain level based on VAS pain for the individual approach groups at the different points in time

*DAA* direct anterior approach; *VAS* visual analog scale

^a^DAA patients reported significantly less pain at rest at 6 weeks control than the lateral and posterior approach groups

^b^DAA patients reported significantly less pain during motion at the 1st postoperative day than the lateral approach group

^c^DAA and posterior approach patients reported significantly less pain at motion at the third postoperative day and at 6 weeks than the lateral approach group

### Analysis of pre-versus postoperative mHHS

**Fig. 3:**
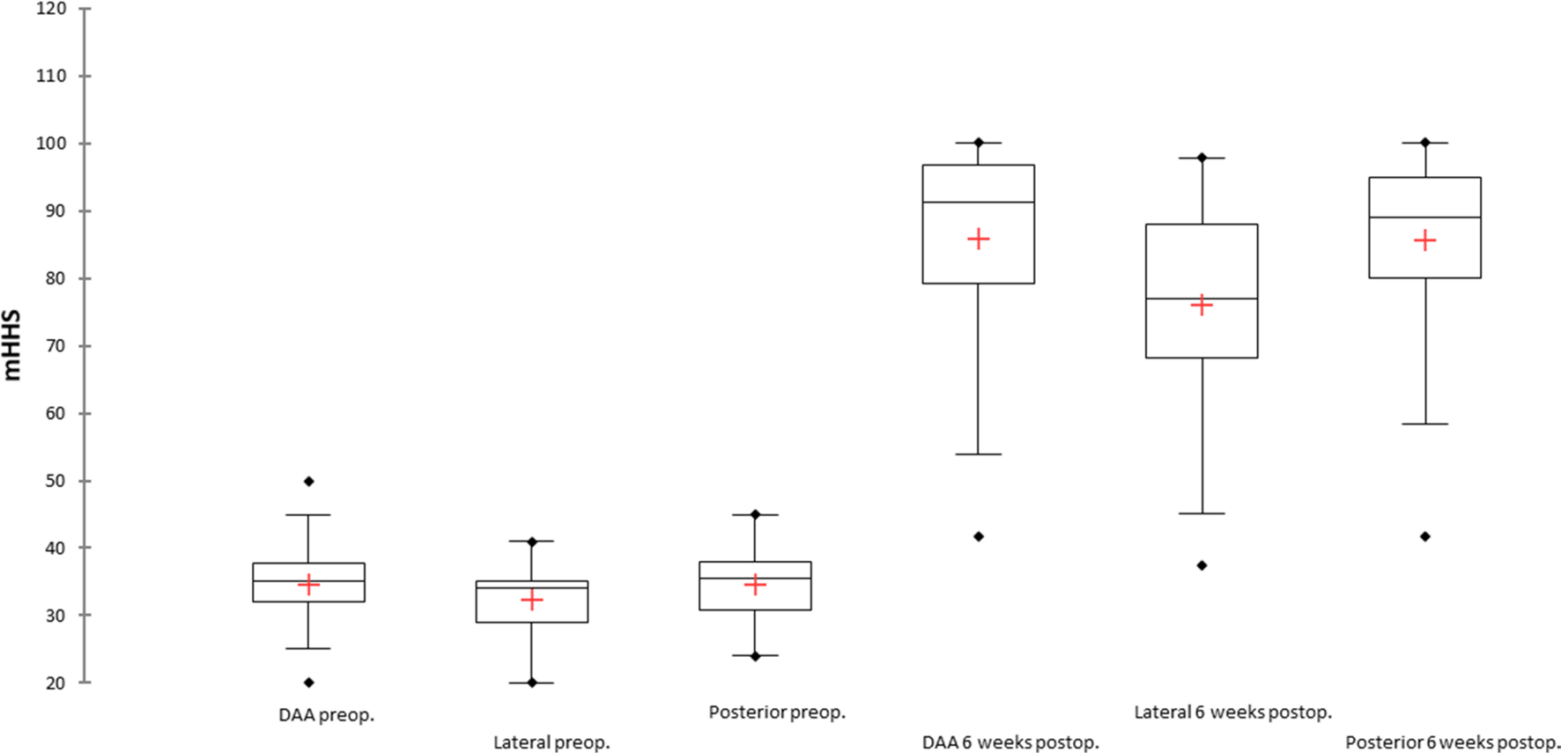
Presentation of the mHHS values for the respective approach groups; comparison of preoperative to 6-week follow-up. *mHHS* modified Harris Hip Score

The data of 177 patients were available for evaluation of the mHHS at the 6-week follow-up. The mHHS demonstrated statistically significant improvement within each group: group 1: preoperative 34.8 ± 4.8 (20.0–50.0) vs. follow-up 85.5 ± 14.6 (41.8–100.0), *p* < 0.00001; group 2: preoperative 32.4 ± 4.7 (20.0–41.0) vs. follow-up 76.2 ± 15.8 (37.4–97.9), *p* < 0.00001; group 3: preoperative 34.8 ± 4.9 (24.0–45.0) vs. follow-up 85.4 ± 13.5 (41.8–100.0), *p* < 0.00001) (Fig. 3).

**Fig. 4:**
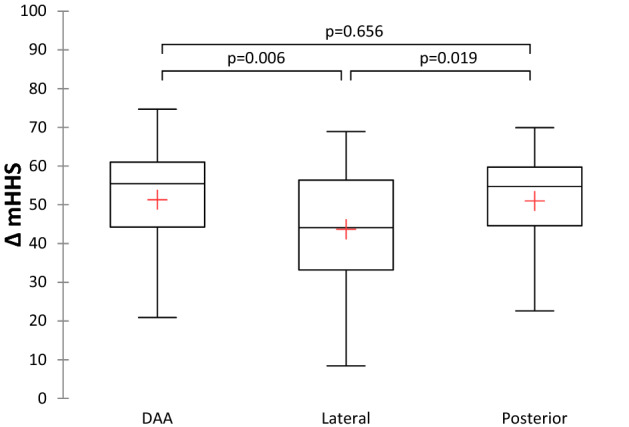
Presentation of the Δ in the mHHS for each group. *DAA* direct anterior approach; *mHHS* modified Harris Hips Score

A significant difference was found among the three groups regarding the respective change in the mHHS value (*p* < 0.033, ANOVA). In the post hoc pairwise comparisons (Bonferroni correction), we found statistically significant differences between groups 1 and 2 (*p* = 0.006) and groups 2 and 3 (*p* = 0.019). No significant differences were found when comparing groups 1 and 3 (*p* = 0.656). The lateral approach group showed significantly less improvement (Fig. 4).

## Discussion

This study analyzed the influence of the surgical approach on perioperative pain and short-term rehabilitation after THA implantation. The main finding was that DAA patients reported the least pain on the third postoperative day and at the 6-week follow-up. In addition, patients treated with a DAA and posterior approach achieved a significantly longer walking distance and thus faster mobilization during the inpatient stay. Patients operated on with the lateral approach achieved the least improvement in the mHHS after 6 weeks. This is significant because, to the best of our knowledge, this is the first study comparing the perioperative effect of three different surgical approaches for THA.

In modern endoprosthetics, the aim is to damage as little soft tissue as possible, which enables faster mobilization and rehabilitation. Most of our patients could already be mobilized on the day of surgery. For the first 2 postoperative days, there were no significant differences in the walking distance achieved in the group comparison. However, patients with the DAA and posterior approach had a significantly longer walking distance on the third postoperative day than those with the lateral approach. Thus, patients who underwent surgery with a minimally invasive approach achieved a longer walking distance by the discharge day. These results are in contrast with Barrett et al.’s study, which compared the anterior and posterior approaches. The DAA subjects walked further postoperatively on the day of surgery and the first and second days postoperatively [25]. The reason for these different results may be due to the surgical technique used for the posterior approach. In our study, the piriformis tendon was not detached and preserved, whereas in Barrett's study, this was not explicitly mentioned. Martusiewicz et al. demonstrated the same walking distance on the discharge day for the anterior and posterior approaches [26].

Another factor influencing perioperative mobilization is pain. Patients operated on with the DAA had significantly less pain at rest than the lateral and posterior approach patients at the 6-week follow-up. Regarding pain during motion, patients with the DAA and posterior approaches had significantly less pain on the third postoperative day and at the 6-week follow-up than patients with the lateral approach. The abductor complex is a pain generator following the direct lateral approach and this may explain differences in early pain perception between the groups [27]. A recent study by Seah et al. investigated postoperative pain and subsequent opioid consumption between three surgical approaches in patients undergoing primary elective THA [16]. In their study, DAA was associated with lower daily opioid usage and pain scores after elective THA in the early postoperative period. Our results confirm these findings, whereby the posterior approach also showed significantly less pain during motion at the third postoperative day and at 6 weeks than the lateral approach. However, our results are in contrast with other studies, that reported lower pain severity for DAA compared to the posterior approach [9, 11, 12]. Nevertheless, the authors could not identify any reason for this and called it speculative [9].

Certain comorbidities have been identified in the literature that can negatively affect postoperative outcomes, especially pain. These factors include diabetes, depression, and chronic pain syndrome [20–23]. In our cohort, there were no significant differences in the distributions of these factors across the groups. Therefore, we assume that these factors did not influence the outcome of the present study. Another possible factor that may influence the perioperative outcome is the duration of surgery. In our cohort, the operation time was significantly longer in the lateral approach group than in the DAA and posterior approach groups. However, it has recently been shown that the duration of surgery has no influence on the postoperative outcome [28], so we do not postulate it had a significant effect on the study outcome. The average operation times in our study were below the reported average operation time for a THA of 93 min [29].

Our study surveyed the mHHS as a PROM. We showed that the lateral approach resulted in a significantly lower improvement than the DAA or the posterior approach. The DAA and posterior approach showed similar improvements at the 6-week follow-up. These findings are consistent with other studies that found less improvements in PROMs for the lateral approach at short-term follow-up [17, 30]. These studies also showed no difference in the short-term results in the PROMS when comparing the DAA with the posterior approach [11, 17].

### Strengths and limitations

One strength of this study is its prospective nature and the inclusion of a consecutive series of patients who received THA. Another advantage is that each surgery was performed by high-volume surgeons, each of whom had a high level of experience in THA.

Our study is not free of limitations. First, we only report a short follow-up period of 6 weeks, so the consistency of the results cannot be assessed. Follow-up studies must, therefore, determine whether the differences found will have persisted. Second, the procedures were carried out by several surgeons, so that individual influences cannot be excluded, and therefore, a surgeon-related bias may exist. However, surgeon selection bias was likely not a factor, as each surgeon performed only the assigned approach during the study period. The lateral approach was performed by only one surgeon, so this group had a small size, which may have influenced the results.

## Conclusion

This study analyzed perioperative pain progression and short-term rehabilitation after THA according to the different surgical approaches. Direct anterior and posterior approaches have shown comparable improvements in pain, walking distance, and mHHS. Whether this effect persists over a longer period of time must be clarified in future studies.
